# Change in Autumn Vegetation Phenology and the Climate Controls From 1982 to 2012 on the Qinghai–Tibet Plateau

**DOI:** 10.3389/fpls.2019.01677

**Published:** 2020-01-15

**Authors:** Peng Li, Qiuan Zhu, Changhui Peng, Jing Zhang, Meng Wang, Junjun Zhang, Juhua Ding, Xiaolu Zhou

**Affiliations:** ^1^ College of Resources and Environmental Science, Hunan Normal University, Changsha, China; ^2^ College of Hydrology and Water Resources, Hohai University, Nanjing, China; ^3^ Center for Ecological Forecasting and Global Change, College of Forestry, Northwest A&F University, Yangling, China; ^4^ Department of Biology Sciences, Institute of Environment Sciences, University of Quebec at Montreal, Succ. Centre-Ville, Montreal, QC, Canada; ^5^ School of Geographical Sciences, Northeast Normal University, Changchun, China

**Keywords:** temperature, precipitation, remote sensing, vegetation dormancy, Qinghai–Tibet Plateau

## Abstract

Autumn vegetation phenology plays a critical role in the survival and reproduction of vegetation in changing environments. Using GIMMS3g (Global Inventory Modeling and Mapping Studies), MODIS (Moderate Resolution Imaging and Spectroradiometer), and SPOT (Systeme Probatoire d’Observation de la Terre) remote sensing data, we investigated the spatial and temporal dynamics of the vegetation dormancy onset date (DOD) and its response to temperature, precipitation, and cold degree days (CDD) in different biomes on the Qinghai–Tibet Plateau (QTP) from 1982 to 2012. Our results indicated that there was no significant temporal trend in the DOD for the vegetation on the QTP but found clear regional characteristics in the DOD trends with a notably advancing trend in the central region and a widespread delay in the southwestern region (>1 day year^−1^, P < 0.05). Our results also indicated that temperature plays an important role in the trend of delays in vegetation autumn phenology; in particular, the preseason temperature can delay the DOD significantly; the positive correlations were observed in more than 71% of the study areas. Consistent with previous studies, we observed significant negative correlations between preseason CDD and DOD; the negative correlations were observed in more than 72% of the study areas for all the data sets. In contrast, the effects of precipitation on DOD were biome dependent. We found that precipitation could promote the extension of the growing season in meadow and grass biomes but produce weak effects on vegetation dormancy in forest biomes. Therefore, not only the magnitude but also the timing of changes in temperature and precipitation determines the effects of climate factors on DOD and further suggests that biome-specific phenological responses also need to be integrated into vegetation phenology models for future climate change investigations on the QTP.

## Introduction

Land surface vegetation plays an essential role in regulating the biosphere and atmosphere by influencing carbon uptake, the hydrological cycle, and energy exchange in ecosystem processes (Visser, 2016). Vegetation phenology refers to the changes in vegetation rhythms caused by the periodic change in ambient conditions (Lieth, 1974), and it is a good proxy of the dynamic response of vegetation ecosystems to climate change (Richardson et al., 2013; Piao et al., 2019). The timing of leaf-out and senescence determines the growing season length of vegetation and plays a critical role in carbon sequestration in vegetation ecosystems (Xie et al., 2015; Tang et al., 2016). Furthermore, changes in vegetation phenology can also affect the interaction of different species and nutrient levels and can even affect the species community compositions (Visser, 2016). Therefore, studies on the land surface vegetation phenology and its response to climate change have drawn increasing attention in global change researches (Piao et al., 2011; Fu et al., 2017; Stucky et al., 2018).

Efforts to investigate the responses of spring phenology to increasing air temperatures have employed ground-based observations of individual trees and regional-scale remote sensing approaches (Fu et al., 2015; Bigler and Vitasse, 2019). Compared with the variations in spring green-up timing, the variations in vegetation dormancy onset date (DOD) and its controlling factors remain less well understood. However, changes in vegetation dormancy could determine the period of vegetation photosynthesis and regulate the carbon balance (Richardson et al., 2013). Evidences from several studies indicated that the extension of the growing season was mainly driven by delayed autumn phenology over temperate and boreal regions in the Northern Hemisphere (Garonna et al., 2014; Fu et al., 2017). Therefore, autumn phenology plays a more direct role than spring phenology in determining net carbon uptake of terrestrial plants. In addition, autumn phenology can also control nitrogen cycling, ecosystem functions, and the associated feedbacks to climate systems (Visser, 2016). Although delayed vegetation autumn dormancy has been reported across a suit of vegetation ecosystem (Gill et al., 2015; Zhu et al., 2017), the potential environmental controls and how they influence vegetation dormancy changes remain poorly understood.

The unique climate conditions on the Qinghai–Tibet Plateau (QTP) play a critical role in the regional ecosystem carbon cycle. Previous studies have reported that the warming climate over the QTP has resulted in significant ecological environmental changes, such as increasing primary production and advancing the spring green-up of vegetation. In particular, the spatiotemporal trend in spring phenology and its response to climate change has been widely studied (Piao et al., 2011; Zhang et al., 2013; Shen et al., 2014; Zhang et al., 2018). However, the vegetation dormancy and associated environmental controlling factors have received less attention on the QTP (Cong et al., 2017). Previous studies intended to investigate the variation of the DOD and its response to the increasing temperature (Ding et al., 2013; Che et al., 2014) while neglecting the role of other climatic drivers. Besides, vegetation composition and climate properties on the QTP exhibits obvious spatial heterogeneity, which result in large discrepancies of DOD response to climatic change (Zhang et al., 2018). Therefore, further investigation of the response of vegetation dormancy to climate change is needed to deepen our understanding of how climate change affects autumn phenology in alpine vegetation on the QTP.

Due to the limitations of ground-based observations for vegetation phenology on the QTP, remote sensing methods have been applied frequently in vegetation phenology monitoring (Piao et al., 2006; Shen et al., 2014). In this study, we examine vegetation autumn dormancy and its relation to temperature and precipitation on the QTP using data from multiple satellites from 1982 to 2012. GIMMS3g (Global Inventory Modeling and Mapping Studies), SPOT (Systeme Probatoire d’Observation de la Terre), and MODIS (Moderate Resolution Imaging and Spectroradiometer) normalized differential vegetation index (NDVI) data sets were used to extract the DOD for the QTP. Because the accumulating cold degree days (temperatures below a base temperature are cumulative after a certain day length, CDD) in growing season have long been considered as the primary triggers of vegetation dormancy (Richardson et al., 2006), we also examined the effects of the base temperatures (10°) on DOD for preseason period. In this study, we aim to (1) systematically examine the spatiotemporal trends of the DOD on the QTP and (2) evaluate the relationship between climate (temperature, precipitation, and CDD) and DOD in different preseason periods and biomes and then explore the underlying mechanisms.

## Materials and Method

### Data Sets

Three NDVI data sets, including GIMMS 3g (1982–2012), SPOT (1999–2012), and MODIS (2000–2012) data sets, were used in our study to extract the DOD. The GIMMS3g data set covered the period 1982–2012 with a spatial resolution of 1/12° and a half-month interval. The MODIS NDVI data set (MOD13C1) for 2000–2012 with a spatial resolution of 0.05°and 16 days’ interval. The SPOT data set was produced every 10 days with a spatial resolution of 0.05° for 2000–2012. These three NDVI data sets have been calibrated for view geometry and have been widely applied for detecting the variation trends in vegetation activity (Shen et al., 2014).

**Figure 1:**
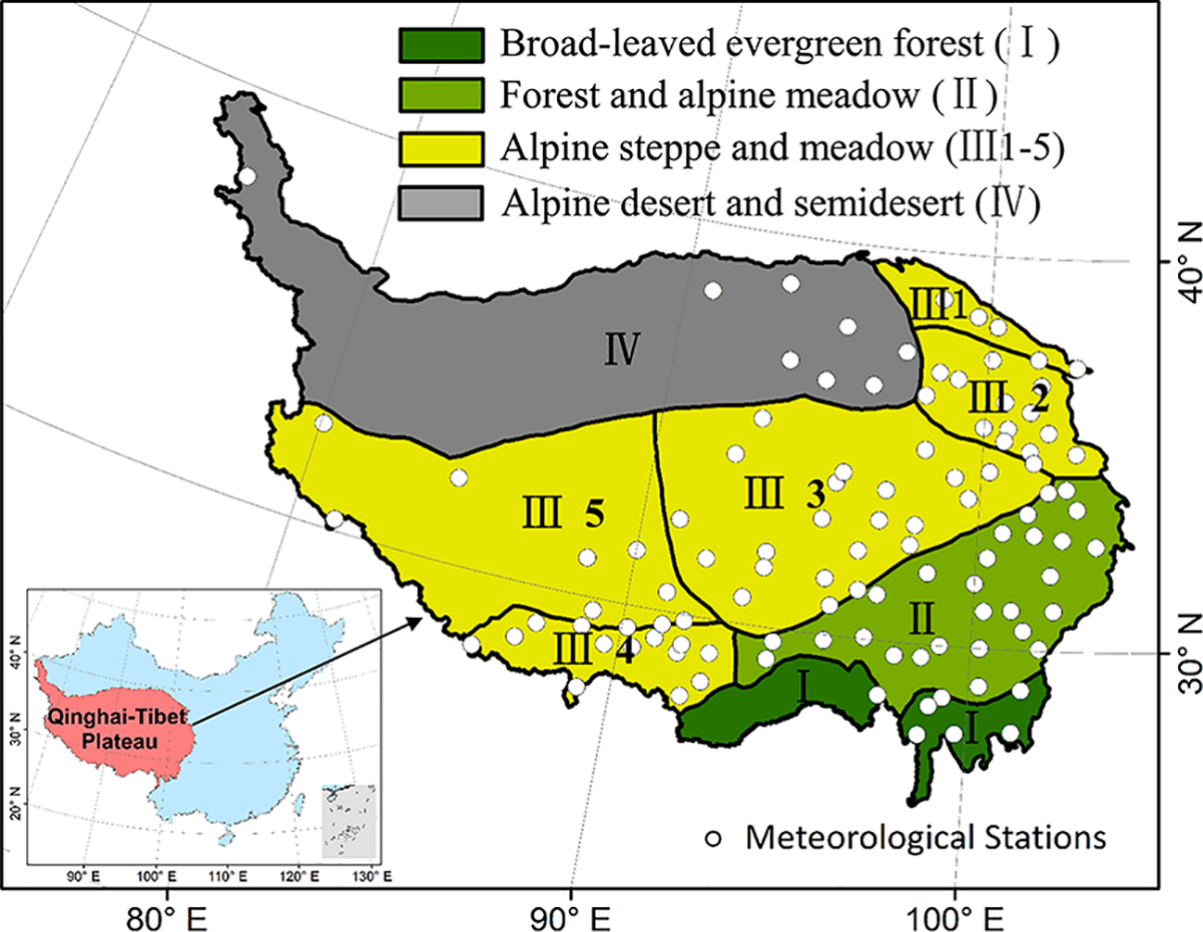
The spatial distribution of meteorological stations and ecological regionalization on the Qinghai–Tibet Plateau (QTP). (Inset) The location of the study region within China. The study area including four ecoregion types: broad-leaved evergreen forests ecoregion (I), forest and alpine meadow ecoregion (II), alpine steppe and meadow ecoregion (III), and alpine desert and semidesert region (IV). The ecoregion III was divided into five subtypes: needle-leaved forest and alpine meadow ecoregion (III 1), agriculture and pasturage ecoregion in eastern Qinghai (III 2), alpine meadow ecoregion (III 3), agriculture and pasturage ecoregion on the southern QTP (III 4), alpine steppe ecoregion (III 5).

Ecological regionalization data were provided for ecoregions in China (Fu et al., 2001), which were taking consideration of the human activities, natural vegetation, and ecological functions. The study region was separated into four ecoregions: broad-leaved evergreen forests ecoregion (I), forest and alpine meadow ecoregion (II), alpine steppe and meadow ecoregion (III), and alpine desert and semidesert region (IV) (**Figure 1**).

We used the climatic data set interpolated from ground-based meteorological record with a spatial resolution of 0.0833° (~10 km) for 1982–2012. Daily meteorological data from 650 meteorological stations were used to construct the climate data sets that include precipitation and air temperature across China with spatial interpolation method of ANUSPLIN, which has great accuracy and applicability in the spatial interpolation of mountain meteorological elements (Xu and Hutchinson, 2013). This meteorological database also has been validated and applied in several studies in China (Zhu et al., 2009; Yang et al., 2016). In our study, gridded daily precipitation and temperature data were extracted from the meteorological database of China.

### Determining the DOD From NDVI Data

Snow cover in non-growing season often distorts the NDVI values, which lead to errors in phenological retrieval (Shen et al., 2013). In our research, we used the daily temperature (below 0°C for 5 consecutive days) to help identify the pixels most likely contaminated by snow and replaced these pixels with the nearest uncontaminated winter NDVI values (Liu et al., 2016). This method has been verified in previous reports (Tan et al., 2011). A smooth curve then was fitted using the Savitzky–Golay filter from the time series of NDVI data (Chen et al., 2004).

Then, the dynamic threshold method and derivative method were applied to extract the DOD from remote sensing time series on the three vegetation indices. A dynamic threshold defined as the NDVI ratio based on the minimum and maximum values of annual NDVI amplitude, which has been widely employed to retrieve the vegetation phenological parameters (White et al., 2009). The NDVI ratio is described as shown in Eq. (1):

(1) $${\text{NDVI}}_{\text{ratio}}=({\text{NDVI}}_{t}-{\text{NDVI}}_{\min})/({\text{NDVI}}_{\max}-{\text{NDVI}}_{\min})$$

Here, NDVI_t_ is the NDVI value at time t; NDVI_max_ and NDVI_min_ stand for the maximum and minimum NDVI values in the annual NDVI time series, respectively. The threshold of 0.6 was originally employed to determine the onset date of vegetation dormancy by Yu et al. (2010) from *in situ* observations on the QTP. To eliminate the influence of sparse vegetation and bare soil on the NDVI, we extracted pixels with an average annual NDVI (1982–2012) greater than 0.1 (Piao et al., 2006).

The second method is the derivative method, and the maximum value of NDVI ratio is the greatest change of NDVI time series. The NDVI ratio is described as shown in Eq. (2):

(2) $${\text{NDVI}}_{\text{ratio}(t)}=\;({\text{NDVI}}_{\left(t\;+\;1\right)}-{\text{NDVI}}_{\left(t\right)})/{\text{NDVI}}_{\left(t\right)}$$

Where NDVI_(t)_ is the NDVI value at time t, NDVI_ratio(t)_ is the calculated relative changing rate of NDVI at time t. The DOD was defined as the days when the smoothed time series had the strongest decrease (Forkel et al., 2015).

### Analyses

The DOD for each pixel was calculated using the multisource remote sensing data on the QTP, including GIMMS3g, SPOT, and MODIS NDVI data sets. Then, the ensemble average annual value of the DOD for the whole study area was calculated from each data set and method. To analyze the interannual trends in DOD in the QTP, we averaged the annual DOD of all pixels on the QTP and obtained the interannual trends in DOD throughout the study area for the three data sets. To detect the trend turning points of DOD during 1982–2012 at the regional scale, the Mann–Kendall (MK) method was used. The MK test is a non-parametric significance test to statistically assess whether there is a monotonic upward or downward trend of a variable overtime (Mann, 1945), which has been applied to multiple trend analysis of NDVI data (Jong et al., 2011; Che et al., 2014). In our study, the sub-periods were divided by those trend turning points at the significance level of 0.05. And the trends of DOD annual changes using the simple linear regression methods were analyzed during these subperiods. For the spatial distribution of the DOD trends, we conducted simple linear regression for each pixel in order to retrieve the temporal trend in DOD. For the MODIS and SPOT data sets, we analyzed only the spatiotemporal change in DOD during 2000–2012.

To evaluate the response of DOD to climatic change in preseason period and biome types, average DOD from a threshold approach and derivative method were used for the NDVI3g, MODIS, and SPOT data sets. The preseason means the periods preceding the DOD date most related to the variation in DOD. In our study, the preseason was defined as the periods (with 1-month steps) before the DOD date (early October) for which the percentage of positive (negative) correlation coefficient between DOD and climatic factors was the highest. The preseason for each of the three climatic factors was determined separately. Subsequently, we applied a temporal partial correlation analysis between DOD and preseason mean temperature, cumulative precipitation, and CDD for all pixels of the QTP. For different biomes, the mean DOD, average temperature, CDD, and cumulative precipitation of each biome were calculated for each year. Then, a partial correlation analysis was applied between the DOD and the corresponding yearly temperatures, cumulative precipitation, and CDD of each biome.

Because the ecoregion I was mainly covered with broadleaved evergreen forest, it is hard to distinguish the changes over the years; thus fewer effective DODs were extracted. For the ecoregion IV (the alpine desert and semidesert region), the sparse vegetation cover also resulted in that fewer effective DODs were extracted in this region. Therefore, we do not evaluate the DOD of these two biomes. In addition, ecoregions III 2 and III 4 were regionalized to agriculture and pasture ecoregions (represented by III 2) because these two ecoregions had the same attributes. Then, an analysis was conducted for the five ecoregions, including II (forest and alpine meadow ecoregion), III 1 (needle-leaved forest and alpine meadow ecoregion), III 2 (agriculture and pasturage ecoregion), III 3 (alpine meadow ecoregion), and III 5 (alpine steppe ecoregion).

## Results

### The Spatial and Temporal Pattern of DOD

**Figure 2:**
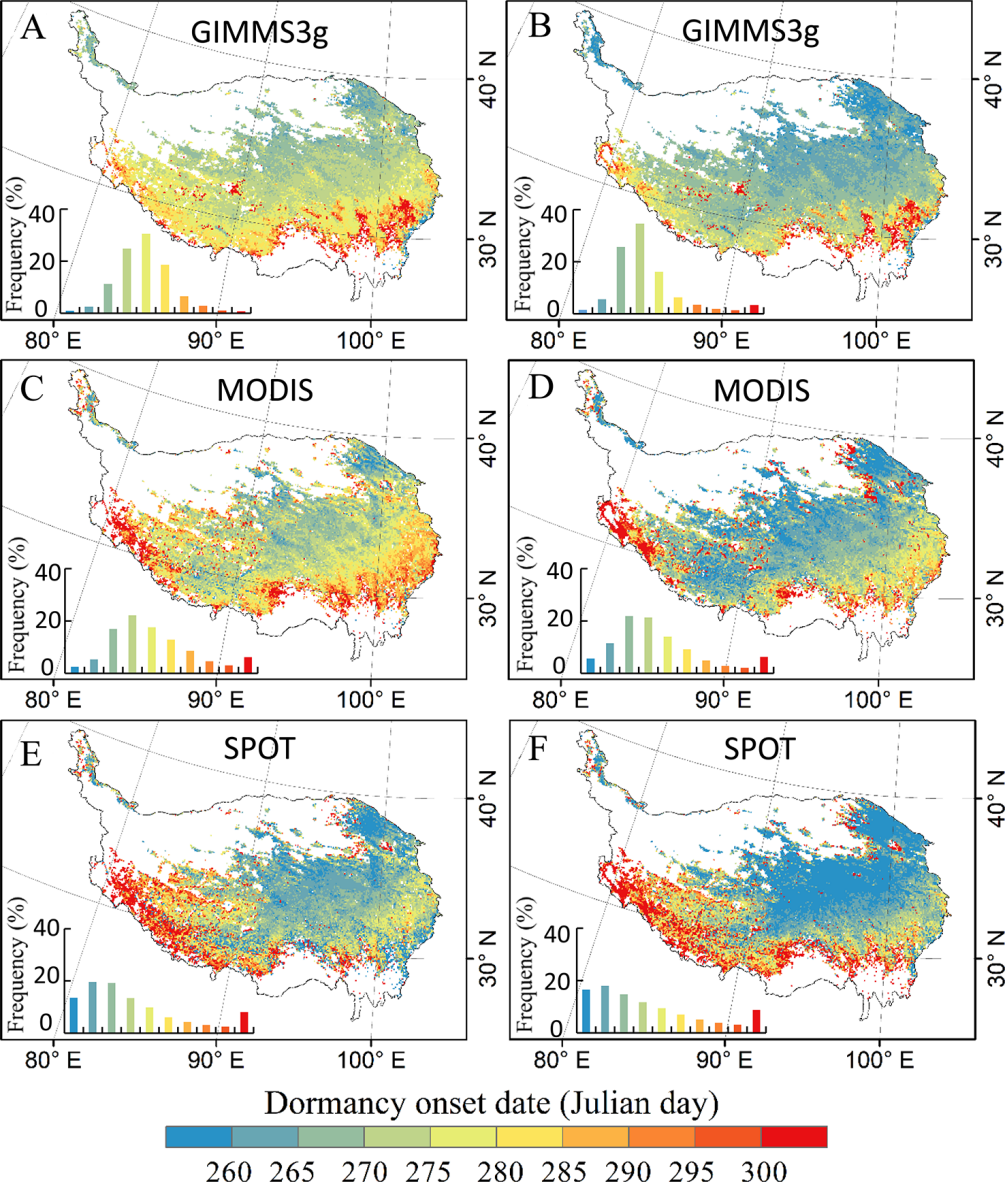
The spatial distribution patterns of the vegetation dormancy onset date (DOD) on the Qinghai–Tibet Plateau based on GIMMS3g (Global Inventory Modeling and Mapping Studies, 1982–2012), SPOT (Systeme Probatoire d’Observation de la Terre, 1999–2012), and MODIS (Moderate Resolution Imaging and Spectroradiometer, 2000–2012) normalized differential vegetation index (NDVI) data sets determined by threshold method **(A, C, E)** and derivative method **(B, D, F)**. The left bottom of each submap displays the DOD frequency distribution in each interval of DOD indicated by the color in the legend in the bottom.

An earlier DOD was found on the northern QTP, whereas the latest DOD was mainly distributed in the southwestern and southeastern regions of the QTP (**Figure 2**). These three data sets showed similar patterns; generally, we found a delaying trend from the central region to the southwest and southeast of the QTP. The frequency distribution of DOD in different data sets showed that more than 90% of the region exhibited an end of vegetation growth between late September and early October. A later DOD was observed in the GIMMS3g and MODIS data sets, while the SPOT data set showed the earliest DOD. The mean DOD values based on threshold method by using the GIMMS3g, SPOT, and MODIS data sets were approximately 275, 265, and 275 days, respectively. Generally, the DOD based on derivative method was more earlier than the result of threshold method for each data set, while the result shows the similar spatial distribution (**Figure 2**).

**Figure 3:**
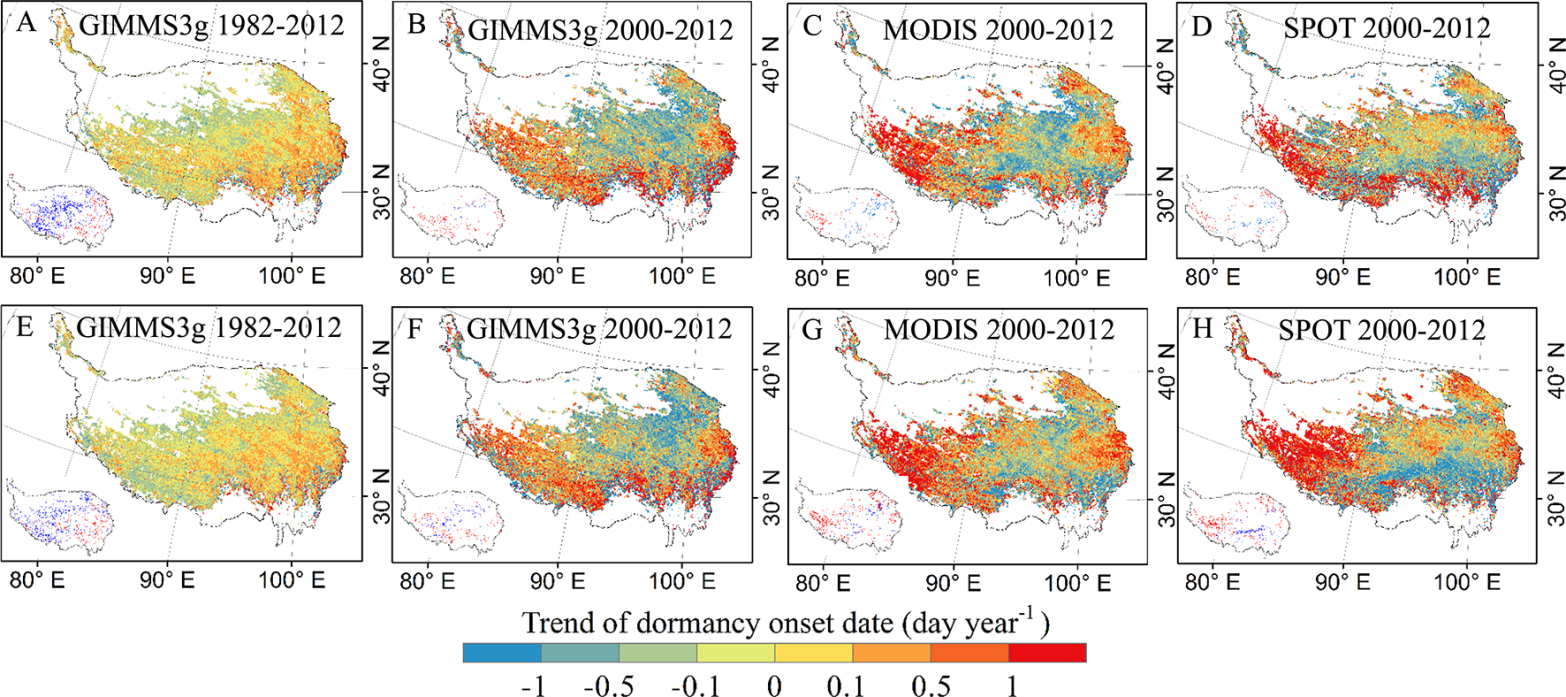
Spatial patterns of trends in the vegetation DOD based on GIMMS3g, SPOT, and MODIS NDVI data sets determined by threshold method **(A–D)** and derivative method **(E–H)**. Pixels with significantly (P < 0.05) positive (red) and negative (blue) trends are shown in the left bottom of each submap.

The spatial patterns of trends in the DOD based on each data set and method are provided in **Figure 3**. DOD of GIMMS3g during 1982–2012 shows a delayed trend in the eastern region of the QTP, while slight advanced trends were observed in the northern and southwest marginal regions (**Figures 3A, E**). For 2000–2012, advancing and delaying trends were observed (**Figure 3**). The great delays are generally concentrated in the southwestern part of the QTP. All three data sets showed a delayed trend from the central to the western and southeastern regions, and a significant delayed trend (>1 day year^−1^, p < 0.05) in DOD was observed in the southwest during 2000–2012 (**Figures 3B–D, F**–**H**). For the different methods, the spatial patterns of trends in the DOD show similar pattern for the three data sets (**Figure 3**).

**Figure 4:**
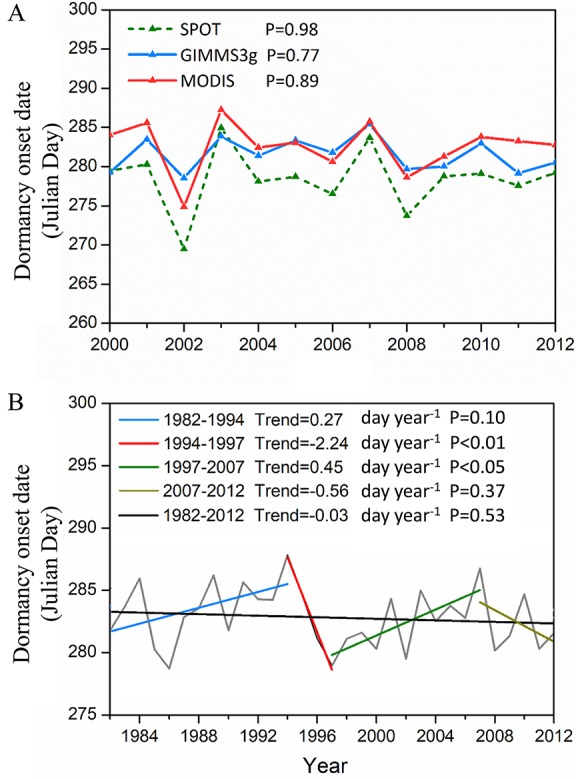
Interannual variations in vegetation DOD. **(A)** Interannual changes in the two methods averaged DOD derived from the GIMMS3g, MODIS, and SPOT NDVI during 2000–2012, respectively. **(B)** Interannual change in two methods averaged DOD derived from the GIMMS3g NDVI. The turning points in the DOD trends (GIMMS3g 1982–2012) determined by the Mann–Kendall test are the years of 1994, 1997, and 2007.

Similar change trends for DOD were observed for the three data sets (**Figure 4**). The trend in DOD was statistically nonsignificant (P > 0.05) for the three NDVI data sets during 2000–2012 (**Figure 4A**). The interannual variations in DOD for the whole study area from 1982 to 2012 were nonsignificant (P = 0.53) (**Figure 4B**). In our study, the DOD trend turning points were calculated using the MK trend test method. The 31 years from 1982 to 2012 were divided into four periods with inflection points of 1994, 1997, and 2007 (**Figure 4B**). The DOD was significantly delayed (slope = 0.27 day year^−1^, P = 0.10) prior to 1994. During the period 1994–1997, the DOD shows a significant advanced trend (slope = −2.24 day year^−1^, P < 0.01). Subsequently, a notable delaying trend was observed for DOD (slope = 0.45 day year^−1^, P < 0.05) until 2007. During the latter period of 2007–2012, the DOD again gradually advanced (slope = −0.56 day year^−1^, P = 0.37).

### Responses of DOD to Climatic Factors

**Figure 5:**
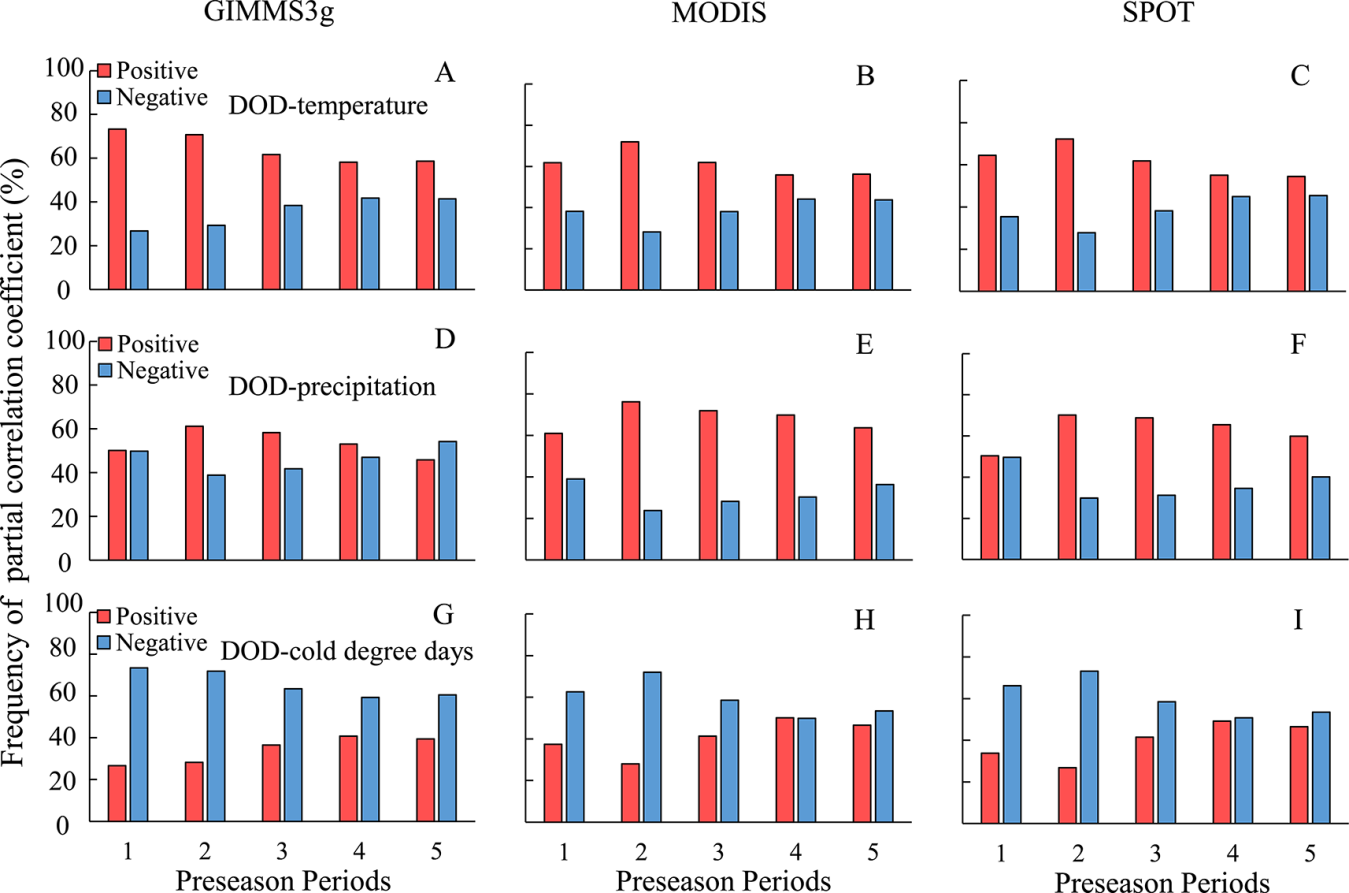
Frequency distribution of the partial correlation coefficient between two methods averaged vegetation DOD of GIMMS3g, MODIS, SPOT, and climatic factors for different preseason periods. **(A–C)** for temperature, **(D–F)** for precipitation, **(G–I)** for cold degree days.

At the regional scale, DOD in QTP was correlated with mean temperature during the periods of 1–5 months prior to DOD; the highest percentage of positive partial correlation coefficient occurred at about 1–2 months for all the three data sets, except for GIMMS3g data (**Figures 5A–C**). In view of the close positive percentage during the periods of 1 (73.3%) and 1–2 months (71.8%) prior to DOD for GIMMS3g data, we uniformly choose the 1–2 months prior to DOD as the preseason length for temperature. With regard to effects of the precipitation and CDD, the periods most associated with DOD both were 1–2 months prior to DOD. The positive relation percentage of DOD and accumulative precipitation in the 1–2 months prior to DOD were observed in more than 61% (**Figures 5D–F**), while the DOD was negatively correlated with CDD during the periods of 1–5 months prior to DOD, the highest negative correlations percentage (more than 74%) were observed in 1–2 months prior to DOD (**Figures 5G–I**).

**Figure 6:**
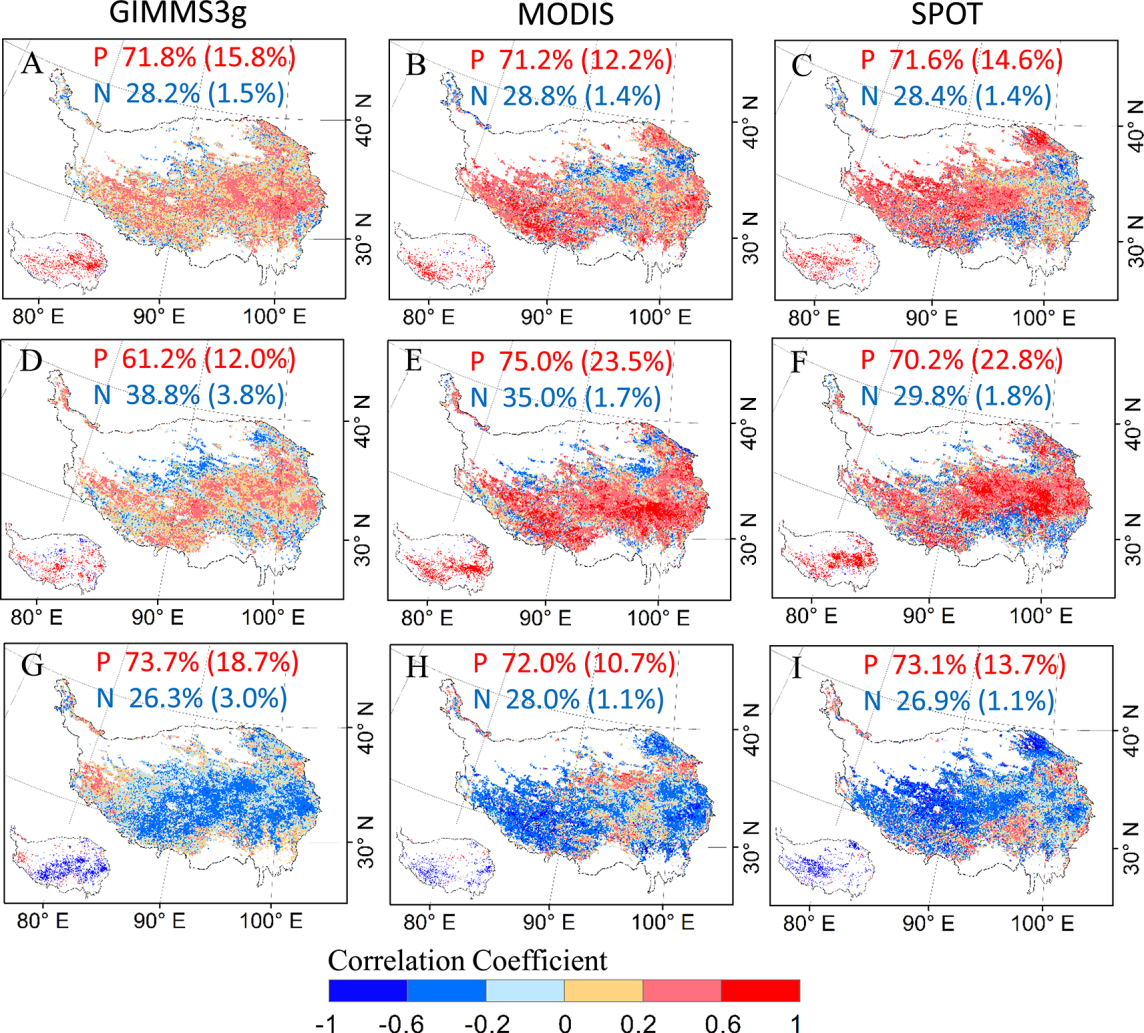
The spatial patterns of partial correlation coefficients between vegetation DOD of GIMMS3g (1982–2012), SPOT (2000–2012), and MODIS (2000–2012) NDVI data sets and climatic factors for preseason period. **(A**–**C)** for the temperature, **(D**–**F)** for the precipitation, **(G**–**I)** for the cold degree days. The inset panels in the bottom left of each submap present the pixels with a significantly (P < 0.05) positive (red) and negative (blue) value. The percentages of positive (P) and negative (N) correlations (percentage of significant correlations in parentheses) are shown at the top of each submap.

In general, our analysis shows a positively relationship between DOD with mean temperature during the preseason for all the three data sets (**Figures 6A–C**). Positive correlations were observed in more than 71% of the study areas; 18% of these correlations were statistically significant (P < 0.05) for GIMMS3g and more than 12% for MODIS and SPOT data sets. A small number of negative correlations account for about 25% of the total pixels, are generally concentrated in the northern and southern marginal regions of the QTP (**Figures 6A–C**). For cumulative precipitation, positive correlations between DOD and precipitation in preseason were observed in 61.2% of the study areas for GIMMS 3g data set, and 12.0% of these correlations were statistically significant (P < 0.05) and were mainly distributed in the central and southwestern regions of the QTP. The percentages of positive correlations between DOD and precipitation reached 75.0% and 70.2% for MODIS and SPOT data set, respectively (**Figures 6D–F**). In contrast to temperature and precipitation, most parts of QTP exhibited a negative relationship between DOD and CDD during the preseason; negative correlations were observed in more than 72% of the study areas for all the data sets; the significant correlations (P < 0.05) account for 18.7%, 10.7, and 13.7 of the total pixels for GIMMS3g, MODIS, and SPOT data, respectively (**Figures 6G–I**).

**Figure 7:**
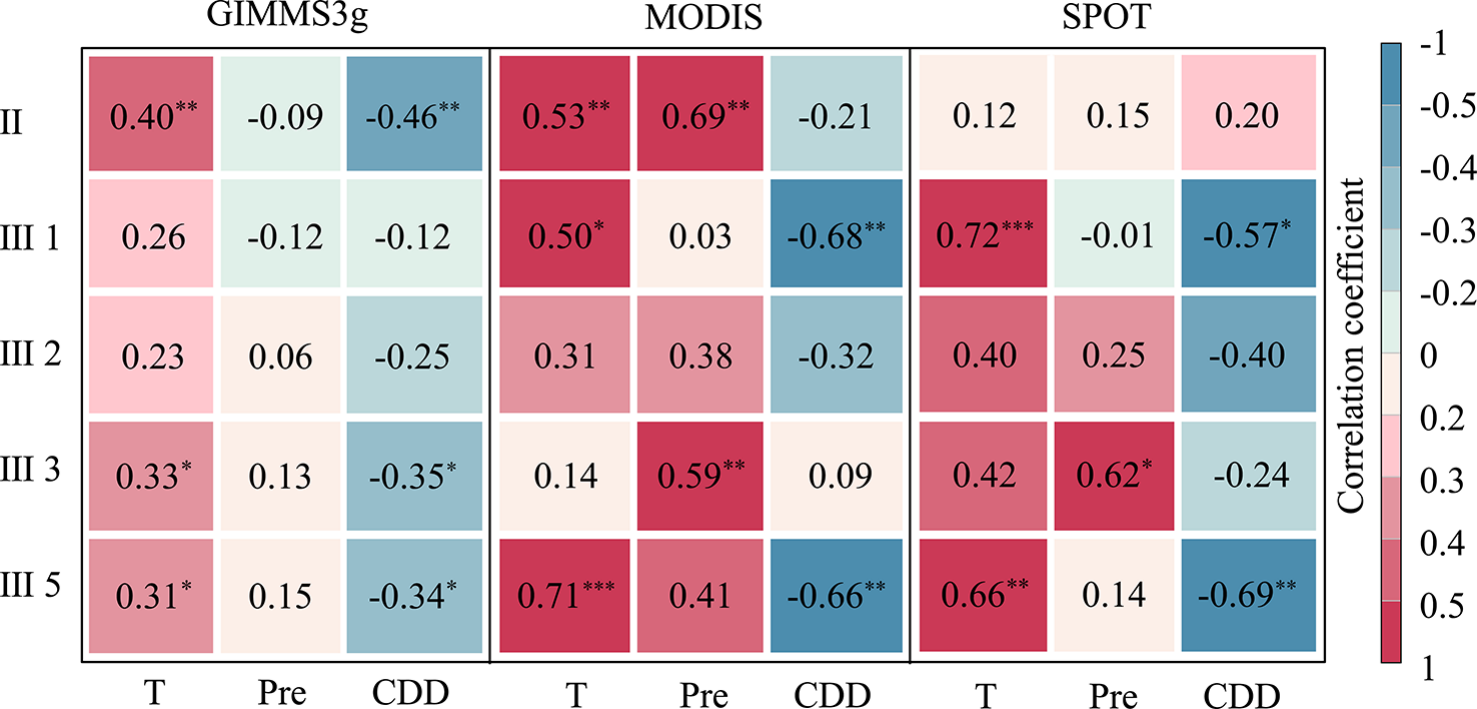
Correlation coefficients between the vegetation DOD of GIMMS3g (1982–2012), SPOT (2000–2012), and MODIS (2000–2012) NDVI data sets and mean temperature (T), precipitation (Pre), and cold degree days (CDD) for different biome types: forest and alpine meadow ecoregion (II), needle-leaved forest and alpine meadow ecoregion (III1), agriculture and pasture ecoregion (III2), alpine meadow ecoregion (III3), alpine steppe ecoregion (III5). ***, **, and * represent the significant correlation at the 0.01, 0.05, and 0.10 levels, respectively. Correlations with no asterisk indicate nonsignificant (P > 0.10).

Consistent positive correlations were observed between DOD and temperature for each biome and data set (**Figure 7**). Specifically, the eastern forest and alpine meadow ecological zone (II), needle-leaved forest and alpine meadow ecoregion in the Qilian Mountains (III1), and alpine steppe ecoregion (III5) were most affected by preseason temperature and exhibited significantly positive correlations for the all three data sets (P < 0.1). In contrast, agriculture and pasture ecoregion (III2) exhibited the least influence of temperature; the weak correlations were observed for the three data sets (**Figure 7**). Compared with the average temperature, the impact of cumulative precipitation on DOD was more complicated, and the partial correlations between DOD and cumulative precipitation were biome dependent. A weak correlation between the DOD and the preseason cumulative precipitation was observed in biome II, III1, and III2, and these correlations were not significant in the preseason (except the MODIS data sets in biome II). For biome III3 and III5, the cumulative precipitation was positively associated with DOD, and the correlations were significant (P < 0.05) in the biome III3 for MODIS and SPOT data sets. For the CDD, a majority of negative correlations were observed between DOD and CDD for each biome and data sets. In biome III3, it shows a significant negative correlation between DOD and CDD in the three data sets, whereas the correlations of other biomes varied in different data sets.

## Discussion

### Change in Vegetation Dormancy on the QTP

Our analysis using remote sensing NDVI time series from multiple sources indicated that there was no significant temporal trend in DOD during the entire research period. Based on NDVI remote sensing data, several previous studies also reported a nonsignificant temporal trend of DOD in the vegetation of QTP (Yang et al., 2015; Li et al., 2018). Our research indicated that opposing trends between the southwestern QTP and other areas lead to nonsignificant temporal trends in the DOD over the entire study area. Spatially, our three sets of remote sensing data also show a delay trend in the DOD at the southwestern QTP, which was in good agreement with Ding et al. (2013), who examined the spatial and temporal trends of the DOD on the QTP during 1999–2009. A previous study revealed a similar shifting pattern in the spring green-up date on the southwestern QTP during the same period (Shen et al., 2014), which implied that the timing of spring phenology can influence autumn phenology dynamics (Keenan and Richardson, 2015). At the regional scale, the trend in the annual changes of the DOD during the period of 1982–1994 (**Figure 4**) is in agreement with the results of Piao et al. (2006), then the DOD experienced a significant advanced trend, which may be caused by the decreasing preseason temperature during this period (Chen et al., 2014). We noticed that the DOD had a delayed trend after 1997; this may due to the large-scale conservation programs implemented by the Chinese government, including the Natural Forest Conservation Program (initiated in 1998) and the Grain for Green Program (initiated in 1999) (Liu et al., 2008), which protect the vegetation from interference of human and lead to an increase in vegetation cover. Because the increase of vegetation cover can increase the amount of solar radiation absorbed by the surface through reduced albedo, which could produce a climate warming effect (Pearson et al., 2013), resulting in a delayed vegetation dormancy (Li et al., 2018). The last turning point of 2007 could be explained by the complicated interaction between the increasing temperature and decreasing precipitation during this period. In addition, our research shows a similar spatial patterns of change trend in DOD by different methods. Previous studies also revealed the same trend for the annual phenological metrics using five different remote sensing–based methods despite some differences in the derived vegetation phenology (Shen et al., 2014; Liu et al., 2016), which suggested that the DOD trend could be applicable for the analysis of longer temporal scales.

### Effects of Climatic Factors on DOD

Our study indicated that the increasing preseason temperature would lead to a delayed vegetation autumn dormancy in the QTP. The generally positive relationship between DOD and average temperature in preseason was because increasing temperature in autumn can enhance the photosynthetic activities and reduce the degradation speed of chlorophyll during vegetation senescence (Shi et al., 2014; Liu et al., 2016). Another possible interpretation is that temperature increase in autumn reduces the frost days in autumn and leads to a later date of first frost in autumn (Gill et al., 2015), which reduces the risk of vegetation response to frost damage and results in delayed vegetation dormancy. Our finding of the negative relationship between vegetation dormancy date and CDD can also explain the vegetation growth strategy, which allows entering dormancy early, avoids unfavorable or damaging growing season conditions, and facilitates the diversion of more resources for use in subsequent years, thus maximizing longer-term fitness (Lim et al., 2007; Xie et al., 2015).

For different biomes, our study indicated that vegetation in the alpine meadow ecoregion and alpine steppe ecoregion experience the most impacts of the preseason average temperature, suggesting that warmer preseason temperatures could delay the vegetation growth season in these biomes. However, our results indicated that the autumn vegetation phenology in agriculture and pasturage ecoregions experience fewer impacts of the preseason average temperature. This result could be because the vegetation in this biome was significantly affected by grazing. Previous studies have suggested that moderate grazing could accelerating the recycling of nutrients flows in soil and enhancing the stability of vegetation ecosystems (Louault et al., 1997), thus moderate the impact of climate change on vegetation. Therefore, the DOD in the agricultural biome shows a weak relationship with temperature. Our result also indicated that the correlations between DOD and CDD were not significant in agriculture and pasturage ecoregion for all three data sets. While the alpine steppe ecoregion experiences the most impacts of the preseason CDD, which may be due to the high altitude and low temperature in this biome, suggesting the DOD was more sensitive to temperature in this biome.

Although temperature plays a critical role in vegetation dormancy, precipitation impacts vegetation dormancy, especially in dry climate areas (Liu et al., 2016; Butz et al., 2018). Our research revealed positive effects of preseason cumulative precipitation on autumn vegetation phenology in most parts of the QTP. Multiple studies have shown that the increasing probability of chlorophyll degradation and vegetation mortality under limited water conditions (Vicente-Serrano et al., 2013; Liu et al., 2016). Therefore, increasing preseason precipitation could relieve drought stress and delay the timing of leaf senescence in arid and semiarid regions. However, our results indicated that the positive effect of precipitation on vegetation dormancy tends to decrease with increasing the preseason time (**Figures 5B, E, H**). This phenomenon indicates that the impacts of precipitation on phenology may be related to precipitation timing and intensity. Our study indicated that the effects of precipitation on DOD varied largely across different biomes. It shows a positive relationship in meadow and grass biomes, whereas a weak relationship was observed in forest biomes. Liu et al. (2016) also revealed a positive effect of precipitation on vegetation dormancy; however, this positive correlation was not observed in forest biomes in temperate China. Two factors may account for this phenomenon. First, cloud cover increases along with increased precipitation, which result in a lower incoming solar radiation (Yang et al., 2015). Second, the soil moisture content is relatively high in forest biomes. Excess moisture input would result in an anaerobic environment within the plant root zone and thus inhibit vegetation growth (Yang et al., 2015). Therefore, increased preseason precipitation would produce more obvious effect on grass biomes.

### Uncertainties and Limitations

Though it showed similar patterns in the three data sets, some difference also persists among sensors. The difference in spatial and temporal resolution of the three data sets will produce some influences on the retrieved DOD. On the one hand, the return interval of different satellite varied from 10 days to 16 days will result in the bias. For example, previous study shows the accuracy of phenology retrieve will be decreased significantly if NDVI values are missing around the onsets of phenological transition dates in the time series of 16-day MODIS data (Zhang et al., 2009). On the other hand, the temporal resolution of remote sensing data could also influence the observed DOD. One pixel for SPOT corresponds to 100 pixels of GIMMS3g in our study, particularly in areas with large changes in terrain and vegetation composited for the 100 pixels, which will result in large difference for the result. For example, in the mixed pixels of the vegetation area and nonvegetated area, the NDVI value of low-resolution images is lower than NDVI value of high-resolution image (Xia et al., 2013), which resulted in large amplitude of annual NDVI time series, and the corresponding extracted DOD was relatively early. This may explain why the latest DOD was found in SPOT data sets.

We also noticed the difference in the response of DOD to different climate factors (**Figure 6**), which indicated that the main control factors of DOD varied from different region, so we should take the biome-specific phenological responses to climate factors into consideration. Our research focuses on the effect of single climate factor on DOD, and ignores the combined effect of precipitation and temperature. So the same attention should be paid to the interactions between temperature and precipitation. Furthermore, recent studies indicated the preseason maximum daytime and minimum nighttime temperatures produced contrasting effects on the DOD (Yang et al., 2017; Wu et al., 2018). So, in order to improve the accuracy of vegetation phenology modeling, the daytime and nighttime temperatures should be incorporated in autumn phenology models, rather than mean temperature alone in future study. Besides climate factors, the vegetation dormancy was also controlled by many other factors, such as spring phenology, and human activities (Yang et al., 2015; Zu et al., 2018). Therefore, future research should also take the physiological responses of vegetation dormancy to multiple environmental controls into consideration, including the interactions among stresses and nonlinear effects.

## Conclusions

Based on GIMMS3g, SPOT, and MODIS NDVI time series, we observed a significant delaying trend in the southwest region and an advanced trend in the central regions of the QTP. Our results indicated that preseason temperature plays a positive role in delaying vegetation dormancy on the QTP. The positive effect of temperature on autumn phenology was weakened as the preseason periods increased. In comparison with temperature, the effects of precipitation on vegetation dormancy are dependent on if the studied region is water limited. The cumulative precipitation in the 2 months prior DOD could promote the delay trend of DOD in most of the study area. For meadow and grass biomes, precipitation could promote the extension of the vegetation growing season. In contrast, for forest biomes, the effect of precipitation on vegetation dormancy is weak. Our study indicated that apart from the magnitude, the timing of changes in temperature and precipitation also affects the response of vegetation dormancy to climate changes. Furthermore, the biome-specific phenological responses to climate factors are also suggested to be taken into consideration in the prediction model of vegetation phenology.

## Data Availability Statement

All datasets for this study are included in the article/supplementary material.

## Author Contributions

PL, CP, and QZ conceived and designed the study. JiZ, MW, JuZ, JD, and XZ processed and analyzed the data. PL wrote the first draft of the manuscript. CP and QZ provided additional advice on the analysis. All authors contributed to manuscript revision, and read and approved the submitted version.

## Funding

This study was financially supported by the National Natural Science Foundation of China (41901117), the Outstanding Youth Project of Hu’nan Provincial Education Department (18B001), and the Natural Sciences and Engineering Research Council of Canada (NSERC) Discover Grant.

## Conflict of Interest

The authors declare that the research was conducted in the absence of any commercial or financial relationships that could be construed as a potential conflict of interest.
